# The Effectiveness of Remote Exercise Rehabilitation Based on the “SCeiP” Model in Homebound Patients With Coronary Heart Disease: Randomized Controlled Trial

**DOI:** 10.2196/56552

**Published:** 2024-11-05

**Authors:** Dandan Xu, Dongmei Xu, Lan Wei, Zhipeng Bao, Shengen Liao, Xinyue Zhang

**Affiliations:** 1 Department of Cardiology The First Affiliated Hospital of Nanjing Medical University Nanjing China

**Keywords:** coronary heart disease, exercise rehabilitation, promotion strategy, home rehabilitation

## Abstract

**Background:**

While exercise rehabilitation is recognized as safe and effective, medium- to long-term compliance among patients with coronary heart disease (CHD) remains low. Therefore, promoting long-term adherence to exercise rehabilitation for these patients warrants significant attention.

**Objective:**

This study aims to investigate the impact of remote exercise rehabilitation on time investment and related cognitive levels in homebound patients with CHD. This study utilizes the SCeiP (Self-Evaluation/Condition of Exercise-Effect Perception-Internal Drive-Persistence Behavior) model, alongside WeChat and exercise bracelets.

**Methods:**

A total of 147 patients who underwent percutaneous coronary intervention in the cardiovascular department of a grade III hospital in Jiangsu Province from June 2022 to March 2023 were selected as study participants through convenience sampling. The patients were randomly divided into an experimental group and a control group. The experimental group received an exercise rehabilitation promotion strategy based on the “SCeiP” model through WeChat and exercise bracelets, while the control group followed rehabilitation training according to a standard exercise rehabilitation guide. The days and duration of exercise, levels of cardiac rehabilitation cognition, exercise planning, and exercise input were analyzed before the intervention and at 1 month and 3 months after the intervention.

**Results:**

A total of 81 men (55.1%) and 66 women (44.9%) were recruited for the study. The completion rate of exercise days was significantly higher in the experimental group compared with the control group at both 1 month (t145=5.429, *P*<.001) and 3 months (t145=9.113, *P*<.001) after the intervention. Similarly, the completion rate of exercise duration was significantly greater in the experimental group (t145=3.471, *P*=.001) than in the control group (t145=5.574, *P*<.001). The levels of autonomy, exercise planning, and exercise input in the experimental group were significantly higher than those in the control group at both 1 month and 3 months after the intervention (*P*<.001). Additionally, the experimental group exhibited a significant reduction in both process anxiety and outcome anxiety scores (*P*<.001). Repeated measures ANOVA revealed significant differences in the trends of cognitive function related to cardiac rehabilitation between the 2 patient groups over time: autonomy, F1,145(time×group)=9.055 (*P*<.001); process anxiety, F1,145(time×group)=30.790 (*P*<.001); and outcome anxiety, F1,145(time×group)=28.186 (*P*<.001). As expected, the scores for exercise planning (t145=2.490, *P*=.01 and t145=3.379, *P*<.001, respectively) and exercise input (t145=2.255, *P*=.03 and t145=3.817, *P*<.001, respectively) consistently demonstrated superiority in the experimental group compared with the control group at both 1 and 3 months after the intervention. Interestingly, we observed that the levels of exercise planning and exercise input in both groups initially increased and then slightly decreased over time, although both remained higher than the preintervention levels (*P*<.001).

**Conclusions:**

The remote health intervention based on the “SCeiP” model effectively enhances exercise compliance, exercise planning, exercise input, and cognitive levels during cardiac rehabilitation in patients with CHD.

**Trial Registration:**

Chinese Clinical Trial Registry ChiCTR2300069463; https://www.chictr.org.cn/showproj.html?proj=192461

## Introduction

### Background

Exercise rehabilitation is a core component of cardiac rehabilitation and plays a vital role in the secondary prevention of coronary heart disease (CHD). It has been shown to significantly reduce adverse cardiovascular events and improve disease prognosis [1]. The center-based rehabilitation model is particularly recognized for its safety and effectiveness in achieving these outcomes [2]. However, the implementation of exercise rehabilitation is often hindered by traffic and medical conditions. Consequently, long-term exercise rehabilitation predominantly occurs in home settings, which presents challenges in ensuring compliance and effectiveness [3]. Home-based rehabilitation typically relies on remote coaching, involving indirect exercise performance, while supervision is conducted in a convenient and acceptable manner [4]. Existing systematic reviews indicate that there are no statistically significant differences in readmission rates or all-cause mortality when comparing the safety profiles of traditional center-based cardiac rehabilitation with those of home-based cardiac rehabilitation [5,6]. However, the participation rate in exercise rehabilitation remains low. Of the approximately 50% of countries worldwide that offer cardiac rehabilitation, only 30% of patients with heart disease participate in these programs [7]. Furthermore, the participation rate for patients with CHD in the general population ranges from 8% to 34% [8], while dropout rates vary from 12% to 56% [9]. Overall, the rate of participation in exercise rehabilitation remains low [10,11]. When extrinsic structural barriers, such as work obligations or scheduling conflicts, are removed [12], it becomes evident that the low participation rate and poor adherence to exercise rehabilitation are likely influenced by a lack of motivation and awareness regarding long-term rehabilitation. This complacency diminishes awareness of the importance of rehabilitation and negatively impacts exercise behavior. The in-depth analysis of adherence to exercise rehabilitation identified 2 main types of influencing factors [13]: self-related factors, which include age, sex, work status, economic conditions, and health knowledge [14]; and external health system factors, which encompass the medical environment, health care personnel, rehabilitation models, and social support [15]. Specifically, patients’ lack of awareness regarding the benefits of exercise rehabilitation, coupled with a fear of pain, has contributed to decreased adherence to exercise [16,17]. In 1998, Johnson and Heller [18] proposed that perceptions of risk factors and benefit factors are significant predictors of behavior. The benefit-risk analysis model [19] further suggests that behavioral intention is closely linked to an individual’s perception of risk and benefit. The level of risk-benefit perception is influenced by various interfering factors, offering new insights into how internal and external factors impact behavioral intention. Classical behavioral theories indicate that generating behavioral motivation is a crucial prerequisite for individual behavior [20,21]. According to social exchange theory, exercise motivation arises from perceptions based on benefits and risks. The formation of accurate perceptions is linked to the appropriate assessment of both internal and external conditions [22].

Previous approaches to behavioral promotion have primarily relied on classical research and analytical methods in psychology and sociology [23]. This reliance somewhat restricts the scope of research on behavioral promotion to existing frameworks, hindering the implementation of promotional measures and the quantification of their mechanisms of action. Building on classical theories in psychology and sociology, Wang [24] proposed the “SCeiP” (Self-Evaluation/Condition of Exercise-Effect Perception-Internal Drive-Persistence Behavior) model. This model emphasizes the self-evaluation of healthy behavior and exercise conditions. It integrates the concepts of effect perception, internal drive, and persistence behavior, forming a transmission mechanism that encompasses perception input, decision-making, motivation, and action output. This mechanism yields positive outcomes for exercise persistence (Multimedia Appendix 1; also see [24]). Based on the “SCeiP” model, this study developed a promotion strategy for exercise rehabilitation behavior and examined its effects on exercise planning, commitment, and adherence among individuals with CHD.

### Objectives

To implement and evaluate the impact of remote exercise rehabilitation promotion strategies based on the “SCeiP” model, we conducted a randomized controlled trial comparing these strategies with current exercise rehabilitation management methods for patients with CHD in a home setting. The primary indicators we observed included compliance with exercise rehabilitation, encompassing both subjective cognitive levels and objective exercise input. To assess the effectiveness and persistence of the intervention, we measured the relevant indicators before the intervention, as well as at 1 month and 3 months after the intervention, conducting both intergroup and intragroup statistical analyses. We hypothesized was that implementing the exercise rehabilitation promotion strategy based on the “SCeiP” model would lead to modest improvements in exercise input and compliance among homebound patients.

## Methods

### Study Design and Setting

This was a single-center, randomized controlled trial, conducted in accordance with the CONSORT (Consolidated Standards of Reporting Trials) reporting guidelines [25] at all stages (Multimedia Appendix 2; also see [26]). The sample size was calculated to compare 2 sample rates [27] using the following formula: N1=N2=[2(*U_α_*+*U_β_*)^2^*P*(1–*P*)]/(*P*_1_ – *P*_2_)^2^, where the test criterion was α=.05 and the probability of type II error was β=.2. Consequently, *U_α_*=1.96 and *U_β_*=0.84; *P*_0_ and *P*_1_ represent the preintervention and postintervention exercise rehabilitation participation rates, respectively, with *P* = (*P*_0_+*P*_1_)/2. Based on preexperimental findings from a small sample, we derived *P*_0_ as 46% and *P*_1_ as 72%, resulting in 63 study participants assigned to both the intervention and control groups. Considering a 20% attrition rate, a final sample size of 76 cases per group was established. A random number table was generated using SPSS 25.0 (IBM Corp.), and the enrolled patients were assigned to either the control group or the intervention group by individuals not involved in the study, according to the random number table.

### Participants

The convenience sampling method was used to select patients who underwent coronary interventional procedures between June 2022 and March 2023 at a tertiary hospital in Jiangsu Province. The inclusion criteria for patients were as follows: (1) diagnosed with CHD; (2) aged 18-70 years; (3) no mental disabilities and capable of effective verbal or written communication; (4) classified according to risk stratification criteria for cardiac rehabilitation exercise, specifically patients with low to medium risk; (5) a Barthel index score of ≥60; and (6) voluntary participation in the investigation with signed informed consent. The exclusion criteria for individuals were as follows: (1) presence of combined diseases that are unsuitable for exercise, such as heart valve disease, severe arrhythmia, or osteoarthritis; (2) presence of serious heart, liver, kidney, or other complications, or other severe consumptive diseases; (3) inability to be followed up after hospital discharge; (4) voluntary withdrawal from the study during its course; and (5) inability to participate in the study as planned. The trial was prospectively registered with the Chinese Clinical Trial Registry (ChiCTR2300069463).

### Experimental Group: Remote Exercise Rehabilitation Based on the “SCeiP” Model

According to the “SCeiP” model proposed by Wang [24], 2 intervention pathways were explored. These pathways begin with “self-evaluation of healthy behavior” and “condition of exercise,” connecting the perceived effects of exercise with internal motivation to enhance adherence to exercise behavior. This results in the “SeiP” path during the exercise cognition stage and the “CeiP” path during the exercise promotion implementation stage. The first draft of the program was developed by integrating the characteristics of exercise rehabilitation for patients with CHD. The final draft was then refined through group discussions and validation by a panel of 5 senior nursing specialists, 3 physicians, and 3 cardiologists.

### Cognitive Phase: The “SeiP” Pathway

This stage is primarily conducted in hospitals. Doctors and rehabilitation instructors utilize the exercise self-assessment form to develop exercise rehabilitation programs tailored to each patient’s condition. Additionally, nurses gather information through a lifestyle self-assessment questionnaire to evaluate the patient’s diet, sleep patterns, self-care abilities, and other relevant factors. Nurses use the patient’s health status to enhance their health records based on test indicators. Additionally, a personal input self-assessment form was used to gather information on the amount of time each patient dedicates to exercise daily. This information is collected during a question-and-answer session with the nurses. The assessments mentioned above are completed 3-5 days after the patient’s admission to the hospital, allowing for the establishment of initial records. Based on the patient’s self-assessment, a cognitive intervention program was developed and delivered through group health education and patient communication sessions. These sessions were conducted 3-5 times, each lasting 30-40 minutes, and were supplemented by face-to-face guidance and question-and-answer sessions with the rehabilitation instructor and cardiologist (Multimedia Appendix 3).

### Implementation Phase: “CeiP” Pathway

#### Exercise Conditioning Phase (3-5 Weeks)

First, we assessed the conditions under which patients engaged in out-of-hospital exercise rehabilitation, including the use of external exercise equipment and devices, the exercise environment, and the support system. Individualized exercise programs were designed based on patients’ exercise preferences at various locations. Patients selected exercise equipment based on their personal preferences, including jump ropes, yoga mats, dumbbells, and other tools. The research team supplied patients with exercise bracelets to monitor vital signs, exercise duration, intensity, and energy expenditure, with data automatically uploaded. This approach enhanced the out-of-hospital management of patients with CHD and fostered a supportive exercise environment. The survey primarily focused on establishing WeChat (Tencent Holdings Limited) groups for patients with CHD. Participants were encouraged to complete and upload their exercise logs daily, organize offline exercise sessions, and perform a 6-minute walk test during outpatient follow-ups for any group exercise involving more than 2 participants. Online and offline interactive methods were integrated to enhance physical examinations. Additionally, the support system was strengthened through improved communication among medical staff, peers, and family members. Regular sharing of benefits aimed to increase patients’ motivation. The WeChat group, exercise logbook, and exercise bracelet provided feedback on patients’ exercise behaviors, allowing doctors and rehabilitators to adjust individualized rehabilitation exercise prescriptions, including the form, frequency, and duration of exercise.

#### Maintenance Phase of Exercise Behavior (5-16 weeks)

Based on the rehabilitation exercise program provided by the rehabilitator, a detailed exercise schedule was established. Patients were required to document the type of daily exercise they performed, along with the actual start and end times, in relation to the prescribed schedule. This allowed them to compare their results against the schedule to determine compliance with the exercise regulations. Each patient then recorded their exercises in a logbook, which was uploaded to the WeChat group daily. A return visit register was established, along with regular telephone follow-ups or home visits, with at least one follow-up conducted. The follow-up visits typically occurred at 6, 10, and 16 weeks, each lasting 30-60 minutes. Patients could report any special circumstances at any time through clinic visits or the WeChat group. Each follow-up visit included an assessment of the patient’s basic condition, exercise rehabilitation program, peer co-training, family support, and availability of exercise equipment. After correcting incorrect rehabilitation methods and living habits, timely feedback from the doctor and rehabilitation division was provided regarding unsuitable exercise programs, facilitating easy adjustments. Follow-up visits were recorded in the patient’s rehabilitation exercise book and retained as stubs. If patients had questions about the exercise program, they could request a case consultation appointment through the WeChat group. The rehabilitator and doctor held online question-and-answer sessions every 2 weeks, lasting 20-30 minutes each, to identify current issues and offer one-on-one guidance.

### Control Group

The control group received a prescribed exercise rehabilitation program and was monitored during follow-up. Cardiologists and rehabilitation specialists collaborated to create individualized exercise rehabilitation programs for each patient. Nurses were responsible for implementing the exercise program log schedule. Patients and their families were instructed on how to record the number of exercise sessions and consultations regarding exercise rehabilitation through various channels. (2) During hospitalization, patients received education about their disease and related exercise rehabilitation. This education aimed to enhance their understanding of CHD and the importance of regular exercise. After discharge, patients were further supported with a guidance program focused on the secondary prevention of CHD and exercise rehabilitation. (3) After discharge, regular telephone follow-ups were conducted, and patients attended follow-up visits every 5 weeks for 30-60 minutes. The rehabilitation clinic and cardiovascular outpatient clinic took special circumstances into account, providing feedback and adjustments tailored to each patient’s situation.

### Measures

#### Adherence to Exercise Rehabilitation

When patients received their exercise rehabilitation prescriptions at 1 month and 3 months after the intervention, data on exercise duration and the number of exercise days per week were collected. A daily exercise duration of up to 80% of the theoretical exercise time was considered an indication of completing one day’s exercise prescription. This study analyzed the percentage of participants who completed the exercise program, as well as the total duration and number of days of exercise. The formula for the completion rate of exercise duration was (actual daily exercise time/theoretical daily exercise time) × 100%, and the formula for the completion rate of exercise days was (actual exercise day/30 × 100%). The theoretical daily exercise time was established by the rehabilitation physician and cardiologist, referencing guidelines for cardiac rehabilitation [28]. Additionally, taking into account each patient’s physical condition, the research group formulated an individualized daily exercise rehabilitation prescription that included the theoretical daily exercise duration. The difference in theoretical exercise duration per day between the 2 patient groups was not statistically significant (t_145_=1.40, *P*=.16).

#### Cardiac Rehabilitation Scale

This scale was developed by Micklewright et al [29] and adapted for the Chinese context by Wang et al [30]. The assessment was conducted to evaluate patients’ cognitive levels regarding personalized cardiac rehabilitation. The scale comprised 3 dimensions: process anxiety, outcome anxiety, and autonomy, totaling 18 items. A 5-point Likert scale was utilized, with the sum of the scores for the corresponding items in each dimension representing the score for that dimension. The total Cronbach α for the scale was 0.816, indicating good internal consistency, while the content validity index was 0.96. The scale was validated based on the following criteria.

#### Exercise Planning Scale

The Chinese version of the Runner Exercise Planning Scale was translated by Shen [31] and consists of 9 items divided into 2 dimensions. Each item is rated on a 5-point Likert scale, ranging from “1=not at all sure” to “5=completely sure.” The Cronbach α for each dimension was 0.8832 and 0.8830, respectively, indicating strong internal consistency. A higher score reflects a stronger exercise planning ability among participants.

#### Exercise Input Scale

The scale comprised 4 dimensions: vigor persistence, focus satisfaction, value perception, and participation autonomy, totaling 20 items. A 5-point Likert scale was used, with scores ranging from 1 to 5, indicating “1=not at all conforming” to “5=fully conforming,” respectively. The total Cronbach α for the scale was 0.906 [32], indicating excellent internal consistency.

### Ethical Considerations

The study was designed in accordance with relevant ethical standards for human experimentation and the Helsinki Declaration. The Medical Ethics Committee of Jiangsu Province Hospital reviewed and approved the study protocol (2021-SRFA-329). All enrolled patients provided informed consent and participated voluntarily. Patient data were used exclusively for this study. The personal information of patients was kept confidential throughout the entire study. After the conclusion of the research, the data were securely sealed without requiring additional consent for secondary analysis. Each participating patient received a complimentary outpatient visit to a specialist in the cardiology department. Additionally, the auxiliary exercise equipment used during the intervention was not removed but left available for continued use by the patients.

### Process and Quality Control Method

The outcomes were measured at 3 time points: baseline (T0: week 0), 1 month after the start of the intervention (T1: week 4), and 3 months after the start of the intervention (T2: week 12). We utilized general information questionnaires to collect data on age, sex, marital status, education level, monthly income, ability to perform activities of daily living, New York Heart Association cardiac function classification, and the number of diseased vessels. Additionally, we assessed the effect of the intervention using the cardiac rehabilitation scale, along with measurements of exercise planning, exercise input, and exercise rehabilitation adherence. Two trained nurses (holding intermediate titles) provided uniform instructions to the participants for completing the scales before the study commenced. Patient data were collected using paper questionnaires and the Questionnaire Star electronic platform at 1 month and 3 months following the intervention. Consistent with previous studies, the dropout rate increased gradually during months 3 and 4 of the exercise rehabilitation process. To prevent omissions, the completion of the questionnaire was verified on-site. At the conclusion of data collection, 2 additional researchers were tasked with entering, checking, and correcting the data without knowledge of the group assignments.

### Statistical Analyses

Data entry and statistical analysis were conducted using SPSS 25.0 software. The quantitative data in this study were normally distributed and presented as mean and SD. To determine whether significant differences existed between groups, the chi-square test was used to examine categorical or nominal variables (eg, gender, age stratification, marital status, education level, residence, income level, cardiac functional grading, and the number of diseased vessels). Additionally, independent samples *t* tests (unpaired, 2-tailed) were performed on continuous variables between the 2 groups, including baseline theoretical average daily exercise duration, completion rates, and score evaluation indicators. A paired samples *t* test was then applied to examine the completion rates of exercise days and duration among the same patients at 1 and 3 months after the intervention. Repeated measures ANOVA was utilized to compare the effects of multiple interactions between group and intervention modality on the levels of cardiac rehabilitation cognition, exercise planning, and exercise input. Differences were considered statistically significant at *P*<.05.

## Results

### Participant Demographics

**Figure 1:**
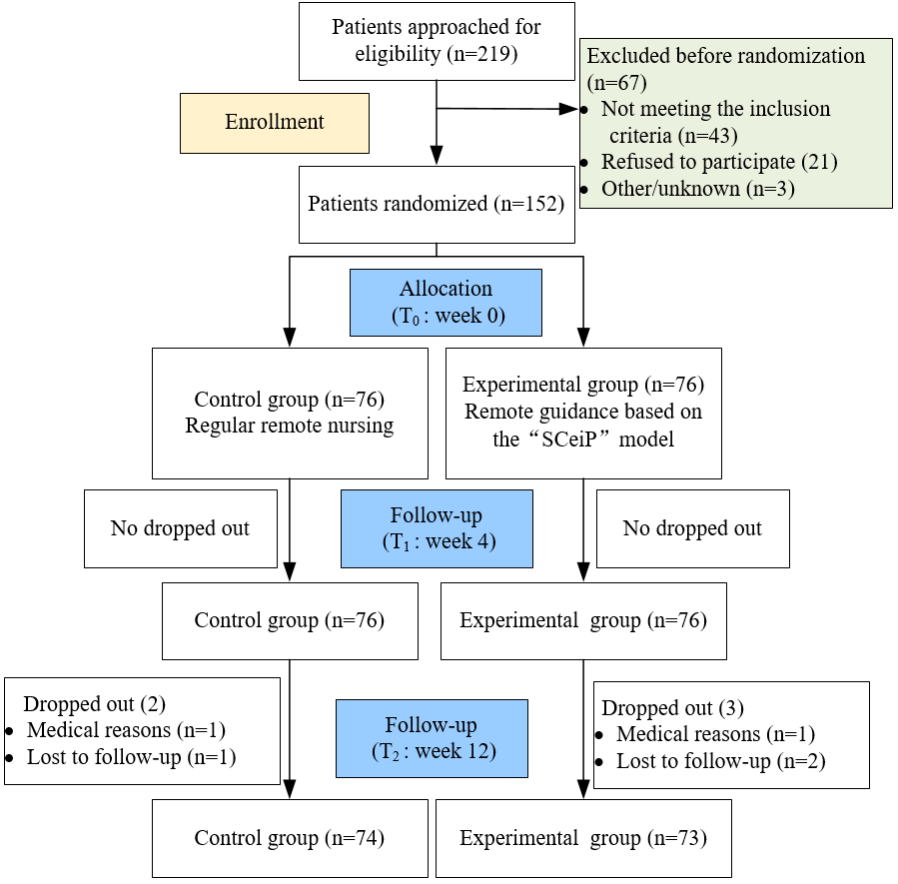
CONSORT (Consolidated Standards of Reporting Trials) flow of participant progression. SCeiP: Self-Evaluation/Condition of Exercise-Effect Perception-Internal Drive-Persistence Behavior.

Out of the initial sample, 5 participants (3.4%) dropped out before the final survey due to medical reasons, including gastrointestinal surgery and neoplastic diseases, as well as loss to follow-up resulting from personal life events. Consequently, a total of 147 participants were included in this study, comprising 81 men (55.1%) and 66 women (44.9%). The participants were divided into an experimental group (n=73, 49.7%) and a control group (n=74, 50.3%; Figure 1). Statistically, there were no significant differences between the 2 groups regarding sex (*P*=.56), age (*P*=.73), marital status (*P*=.70), education level (*P*=.88), place of residence (*P*=.81), personal monthly income (*P*=.75), New York Heart Association cardiac function classification (*P*=.50), number of diseased vessels (*P*=.69), or theoretical exercise duration (Table 1). Additionally, no cardiovascular-related adverse events were reported during the 3-month study period.

**Table 1:** Baseline characteristics of participants.

Variables		Experimental group (n=73)	Control group (n=74)	*χ*^2^/*t* test (*df*)	*P* value
**Gender, n (%)**				0.347^a^ (145)	.56
	Female	31 (42.5)	35 (47.3)		
	Male	42 (57.5)	39 (52.7)		
**Age (years), n (%)**				0.622^a^ (145)	.73
	<30	10 (13.7)	12 (16.2)		
	30-60	48 (65.8)	44 (59.5)		
	>60	15 (20.5)	18 (24.3)		
**Marital status, n (%)**				0.147^a^ (145)	.70
	Married	68 (93.2)	71 (95.9)		
	Single	5 (6.8)	3 (4.1)		
**Education level, n (%)**				0.657^a^ (145)	.88
	Primary and below	17 (23.3)	21 (28.4)		
	Junior high school	25 (34.2)	23 (31.1)		
	Senior high school	14 (19.2)	15 (20.3)		
	College and above	17 (23.3)	15 (20.3)		
**Residence, n (%)**				0.055^a^ (145)	.81
	City	44 (60.3)	46 (62.2)		
	Town	29 (39.7)	28 (37.8)		
**Monthly personal income (RMB^b^), n (%)**				0.579^a^ (145)	.75
	<5000	26 (35.6)	22 (29.7)		
	5000-10,000	37 (50.7)	41 (55.4)		
	>10,000	10 (13.7)	11 (14.9)		
**NYHA^c^, n (%)**				1.385^a^ (145)	.50
	Level 1	46 (63.0)	51 (68.9)		
	Level 2	24 (32.9)	22 (29.7)		
	Level 3	3 (4.1)	1 (1.4)		
**Number of diseased vessels, n (%)**				0.736^a^ (145)	.69
	1	52 (71.2)	48 (64.9)		
	2	19 (26.0)	23 (31.1)		
	3	2 (2.7)	3 (4.1)		
Theoretical average daily exercise duration (minutes), mean (SD)		45.12 (5.15)	43.76 (6.60)	1.401^d^ (145)	.16

^a^Chi-square test.

^b^1RMB=US $0.14.

^c^NYHA: New York Heart Association.

^d^Independent samples *t* test.

### Comparison of Adherence to Exercise Rehabilitation

The completion rates of exercise days declined gradually in both the intervention (t_72_=2.973, *P*=.004) and control groups (t_73_=9.457, *P*<.001). Similar results were observed for the completion rates of exercise duration (t_72_=8.252, *P*<.001 for the intervention group and t_73_=31.054, *P*<.001 for the control group). The results of the intervention study revealed that the strategies based on the “SCeiP” model exhibited significantly higher exercise completion rates at 1 and 3 months after the intervention (t_145_=5.429, *P*<.001) compared with the control group (t_145_=9.113, *P*<.001). Similarly, the completion rate of exercise duration (t_145_=3.471, *P*=.001) was significantly greater than that of the control group (t_145_=5.574, *P*<.001; Table 2).

**Table 2:** Comparison of adherence to exercise rehabilitation between the 2 groups.

Comparison	Completion rate of exercise days, mean (SD)				Completion rate of exercise duration, mean (SD)		
	T_1_	T_2_	*t* test (*df*)	T_1_		T_2_	*t* test (*df*)
Intervention group	67.76 (9.56)	64.38 (9.47)	2.973^a^ (72)	84.01 (5.77)		82.17 (5.51)	8.252^b^ (72)
Control group	58.78 (10.46)	50.09 (9.54)	9.457^b^ (73)	80.58 (6.20)		76.77 (6.22)	31.054^b^ (73)
*t* test (*df*)	5.429 (145)	9.113 (145)	N/A^c^	3.471 (145)		5.574 (145)	N/A
*P*	<.001	<.001	N/A	0.001		<.001	N/A

^a^*P*=.004.

^b^*P*<.001.

^c^N/A: not applicable.

### Comparison of Cognitive Level of Cardiac Rehabilitation

There was no statistically significant difference in the scores for the 3 dimensions of cardiac rehabilitation—autonomy, process anxiety, or outcome anxiety—between the 2 groups of patients before the intervention (Tables 3-5). However, the level of autonomy in the intervention group showed a significant increase compared with the control group at 1 month (t_145_=4.227, *P*<.001) and 3 months (t_145_=6.277, *P*<.001) after the intervention, with a gradual improvement observed over time in both groups. The scores for process anxiety at 1 month (t_145_=–2.950, *P*=.004) and 3 months (t_145_=–6.367, *P*<.001) and for outcome anxiety at 1 month (t_145_=–8.999, *P*<.001) and 3 months (t_145_=–8.424, *P*<.001) exhibited a significant decrease in the SCeiP group. The trends in changes in cognitive function during cardiac rehabilitation differed significantly between the 2 groups over time: autonomy (*F*_1,145[time×group]_=9.055, *P*<.001), process anxiety (*F*_1,145[time×group]_=30.790, *P*<.001), outcome anxiety (*F*_1,145[time×group]_=28.186, *P*<.001; Tables 3-5). This indicates that the implementation of interventions resulted in a gradual improvement in patients’ cognitive levels related to cardiac rehabilitation over time.

**Table 3:** Comparison of the autonomy dimension of Cardiac Rehabilitation Scale scores between the 2 groups.

Comparison	T_0_, mean (SD)	T_1_, mean (SD)	T_2_, mean (SD)	*F*_time_ test (*df*)	*F*_group_ test (*df*)	*F*_time×group_ test (*df*)
Experimental group	12.10 (3.05)	19.01 (2.60)	20.03 (1.97)	461.821^a^ (1, 145)	17.878^a^ (1, 145)	9.055^a^ (1, 145)
Control group	11.74 (3.33)	16.93 (3.32)	17.77 (2.37)	N/A^b^	N/A	N/A
*t* test (*df*)	0.67 (145)	4.227 (145)	6.277 (145)	N/A	N/A	N/A
*P* value	.50	<.001	<.001	N/A	N/A	N/A

^a^*P*<.001.

^b^N/A: not applicable.

**Table 4:** Comparison of the process anxiety dimension of Cardiac Rehabilitation Scale scores between the 2 groups.

Comparison	T_0_, mean (SD)	T_1_, mean (SD)	T_2_, mean (SD)	*F*_time_ test (*df*)	*F*_group_ test (*df*)	*F*_time×group_ test (*df*)
Experimental group	20.60 (3.59)	15.51 (3.59)	12.78 (2.69)	1272.100^a^ (1, 145)	13.408^a^ (1, 145)	30.790^a^ (1, 145)
Control group	21.03 (3.80)	17.26 (3.60)	16.01 (3.41)	N/A^b^	N/A	N/A
*t* test (*df*)	–0.696 (145)	–2.95 (145)	–6.367 (145)	N/A	N/A	N/A
*P* value	.49	.004	<.001	N/A	N/A	N/A

^a^*P*<.001.

^b^N/A: not applicable.

**Table 5:** Comparison of the outcome anxiety dimension of Cardiac Rehabilitation Scale scores between the 2 groups.

Comparison	T_0_, mean (SD)	T_1_, mean (SD)	T_2_, mean (SD)	*F*_time_ test (*df*)	*F*_group_ test (*df*)	*F*_time×group_ test (*df*)
Experimental group	12.41 (2.36)	8.00 (1.87)	6.79 (2.44)	235.586^a^ (1, 145)	72.392^a^ (1, 145)	28.186^a^ (1, 145)
Control group	13.04 (2.56)	11.05 (2.23)	9.95 (2.08)	N/A^b^	N/A	N/A
*t* test (*df*)	–1.549 (145)	–8.999 (145)	–8.424 (145)	N/A	N/A	N/A
*P* value	.12	<.001	<.001	N/A	N/A	N/A

^a^*P*<.001.

^b^N/A: not applicable.

### Comparison of Exercise Planning

The difference in preintervention exercise planning scores between the 2 groups was not statistically significant (*P*=.17). However, the exercise planning scores in the experimental group were significantly higher than those in the control group at both 1 month (t_145_=2.490, *P*=.01) and 3 months (t_145_=3.379, *P*<.001) after the intervention. Interestingly, during the intervention, exercise planning scores for both groups initially increased and then decreased; however, both remained significantly higher than preintervention levels (*F*_1,145[time]_=505.053, *P*<.001). Additionally, the trend of change in exercise planning differed significantly over time between the 2 groups (*F*_1,145[time×group]_=3.527, *P*=.03; Table 6).

**Table 6:** Comparison of exercise planning scores between the 2 groups.

Comparison	T_0_, mean (SD)	T_1_, mean (SD)	T_2_, mean (SD)	*F*_time_ test (*df*)	*F*_group_ test (*df*)	*F*_time×group_ test (*df*)
Experimental group	31.82 (3.77)	36.93 (3.83)	34.68 (3.83)	505.053^a^ (1, 145)	7.503^b^ (1, 145)	3.527^c^ (1, 145)
Control group	30.97 (3.63)	35.36 (3.80)	32.76 (3.05)	N/A^d^	N/A	N/A
*t* test (*df*)	1.392 (145)	2.49 (145)	3.379 (145)	N/A	N/A	N/A
*P*	.17	.01	.001	N/A	N/A	N/A

^a^*P*<.001.

^b^*P*=.007.

^c^*P*=.03.

^d^N/A: not applicable.

### Comparison of Exercise Input Levels

The difference in preintervention exercise input scores between the 2 groups was not statistically significant (*P*=.68). However, at 1 and 3 months after the intervention, the exercise input scores in the experimental group (t_145_=2.255, *P*=.03) were significantly higher than those in the control group (t_145_=3.817, *P*<.001). Similarly, we observed that the scores for exercise input initially increased and then decreased, yet both remained significantly higher than the preintervention levels (*F*_1,145[time]_=302.616, *P*<.001). Additionally, the pattern of change in exercise input showed significant temporal variation as the duration of the intervention period increased (*F*_1,145[time×group]_=10.886, *P*<.001; Table 7).

**Table 7:** Comparison of exercise input scores between the 2 groups.

Comparison	T_0_, mean (SD)	T_1_, mean (SD)	T_2_, mean (SD)	*F*_time_ test (*df*)	*F*_group_ test (*df*)	*F*_time×group_ test (*df*)
Experimental group	66.07 (9.56)	76.37 (9.20)	74.48 (8.28)	302.616^a^ (1, 145)	4.971^b^ (1, 145)	10.886^a^ (1, 145)
Control group	65.45 (8.94)	72.97 (9.06)	69.47 (7.61)	N/A^c^	N/A	N/A
*t* test (*df*)	0.408 (145)	2.255 (145)	3.817 (145)	N/A	N/A	N/A
*P*	.68	.03	<.001	N/A	N/A	N/A

^a^*P*<.001.

^b^*P*=.03

^c^N/A: not applicable.

## Discussion

### Overview

We conducted a randomized controlled trial to evaluate the efficacy of remote exercise rehabilitation based on the “SCeiP” model in patients with CHD. Our study was powered to detect small differences between the exercise rehabilitation strategies while ensuring no adverse cardiovascular events occurred. The findings indicated significant improvements over time in patients’ cognitive levels regarding cardiac rehabilitation, the rate of implementation of exercise rehabilitation, and adherence compared with the traditional exercise rehabilitation model. Moreover, these indicators sustained marked improvement after the intervention.

### Principal Findings

Based on the “SCeiP” model, we implemented an exercise rehabilitation promotion strategy utilizing information technology, including WeChat exercise groups, exercise bracelets, and online consultation applets. This approach enhanced the accuracy and convenience of acquiring knowledge related to cardiac rehabilitation, particularly for patients engaging in home-based exercise rehabilitation. A cross-sectional analysis [33] showed that patients with CHD who highly accepted personalized, user-friendly eHealth platforms with remote monitoring were more willing to maintain phase III cardiac rehabilitation. Additionally, Ramachandran et al [5] reported that the implementation of exercise rehabilitation strategies facilitated by informational tools can effectively enhance patients’ quality of life, improve unhealthy lifestyle habits, and reduce risk factors for adverse cardiovascular events. The reasons for this improvement may include the real-time sharing of exercise prescriptions through WeChat groups, the ability to use keyword searches to obtain methods for monitoring vital signs such as blood pressure and heart rate, the application of techniques for evaluating exercise effects, and the utilization of smart exercise equipment that automatically records exercise results [34]. Moreover, with the assistance of smart exercise equipment, electrocardiograms, metabolic parameters, training intensity, and exercise prescriptions are automatically transmitted to medical personnel, enabling timely adjustments and improvements to exercise prescriptions [35]. Additionally, this smart equipment, combined with online diagnosis and treatment, can help individuals identify abnormal electrocardiograms and arrhythmias during exercise, providing early warnings and interventions related to exercise risks [36]. The implementation of eHealth intervention strategies, including wearable devices, monitoring tools, and web-based portals, has the potential to enhance the efficacy of exercise rehabilitation training [37]. The results indicated that the difference in cardiac rehabilitation–related cognitive status between the 2 groups began to widen at 1 month after the intervention and became more pronounced at 3 months. This suggests that the exercise rehabilitation facilitation strategy used in the experimental group had a lasting impact on improving cardiac rehabilitation cognition in patients with CHD and that its effectiveness progressively outperformed that of the control group. Guo et al [38] also demonstrated that remote rehabilitation can enhance the long-term effectiveness of cardiac rehabilitation for patients. In this study, the combination of WeChat and a smart bracelet facilitated its application and sustained usage.

The exercise rehabilitation strategies emphasized the creation of individualized exercise rehabilitation prescriptions and outcome tracking, offering targeted guidance for both in-hospital phase I and home phase II cardiac rehabilitation for patients with CHD. This approach led to improved levels of exercise planning and input. Nabutovsky et al [39] also found that extended monitoring of prescribed exercise implementation positively influenced patients’ adherence to rehabilitation. This improvement may be attributed to the increased degree of patient participation. The process of formulating precise exercise prescriptions considers the patient’s exercise preferences and adjusts the program based on their actual exercise conditions, thereby enhancing the patient’s ability to engage in collaborative decision-making [40]. Additionally, the external support system was strengthened, as social support from family and health care providers positively influences patients’ knowledge, beliefs, and behaviors, fostering a greater willingness to engage in exercise [41]. Thus, home-based cardiac rehabilitation can enhance patients’ exercise planning and commitment when supported by information technology and personalized exercise program development [42]. This study demonstrated that both groups exhibited a greater willingness to exercise compared with the preintervention period. Moreover, the differences between the 2 groups in terms of exercise planning and input varied significantly over time. The results showed that patients’ exercise planning initially increased, followed by a subsequent decrease; however, both levels remained significantly higher than the preintervention baseline. This indicates that exercise rehabilitation promotion strategies positively impacted compliance, and that interventions based on remote strategies could further optimize patient outcomes. This finding aligns with the study by Lahtio et al [43], which demonstrated that integrating remote technology into cardiac rehabilitation programs significantly enhances positive health outcomes for individuals diagnosed with cardiovascular diseases. Interestingly, the differences in the completion rates of exercise days and duration, as well as in exercise planning and exercise input, were more pronounced 1 month after the intervention. However, over time, these changes appeared to diminish. This may be attributed to the fatigue that often occurs after extended periods of exercise rehabilitation. Our previous qualitative interviews revealed that patients experienced a decline in motor performance during prolonged exercise rehabilitation. Relevant research findings on this topic have been documented and are currently being submitted.

### Strengths and Future Directions

To our knowledge, studies on adherence in exercise rehabilitation have primarily focused on subjective measurement indicators. The evaluation indicators in this study not only include subjective indicators such as cognitive level and exercise planning, but also enhance the objectivity of evaluation indicators by documenting the frequency and duration of each exercise session. Furthermore, it is noteworthy that the introduced “SCeiP” model, which advocates for the preservation of motor behavior, aligns with the purpose of this study. The “SCeiP” model summarizes the internal motivations for exercise behavior as “wanting to do” or “being able to do” (ie, motivation and self-efficacy). These motivations are influenced by the individual’s health, self-evaluation, and perceptions of the conditions and effects of exercise. The model considers “self-evaluation of healthy behavior” as an internal perspective and “condition of exercise” as an external perspective. Ultimately, it encourages patients to make informed exercise decisions and maintain consistent adherence to exercise rehabilitation behaviors. By mediating the perception of exercise effects, the model translates self-evaluation into internal motivation for exercise, optimizes factors associated with exercise behavior, and enhances the synergistic effect of these factors on exercise adherence. This process ultimately increases patients’ inclination to engage in physical activity. The implementation of the SCeiP model in this study enhances compliance with exercise rehabilitation and augments the cardiac benefits for patients with CHD. Previous literature has demonstrated that remote cardiac rehabilitation is advantageous for reducing readmission rates [44] and medication costs [45]. The incorporation of the SCeiP model not only improves participation and sustainability in exercise rehabilitation but also holds significant economic value, warranting further discussion. Moreover, WeChat and exercise bracelets were effectively utilized to promote and supervise the implementation of the SCeiP model. Existing studies have also reported the positive role of smartphone-based programs [46], wearable devices [47], and other information technologies in remote rehabilitation, suggesting that further development and application of artificial intelligence technology may unlock additional possibilities for remote cardiac rehabilitation.

### Limitations

There were several limitations in this study. First, it is imperative to consider expanding the subject enrollment to encompass a larger sample size, with particular emphasis on enhancing exercise rehabilitation compliance among patients with CHD who also have obesity, diabetes, or other chronic diseases. Second, this study exclusively recruited participants from a single university in Nanjing, China, and its generalizability to a larger population remains undetermined. To address this limitation, future research could consider conducting multicenter randomized controlled trials. Third, the intervention period of 3 months could be extended to further observe long-term patient participation in exercise rehabilitation. Lastly, the research measures involve information tools such as WeChat groups and exercise bracelets, which may not fully represent the latest advancements in information technology. Therefore, it is advisable to incorporate new technological approaches, such as wearables, into the formulation of the scheme.

### Conclusions

Based on the “SCeiP” model, this study developed a dual-path practice strategy to enhance exercise rehabilitation in patients with CHD. This strategy integrated both online and offline health education, along with the evaluation of subjective records and objective data that complemented each other. Patients’ adherence to exercise rehabilitation, exercise planning, exercise input, and cognitive levels in remote exercise rehabilitation improved significantly compared with the traditional model, showing better outcomes than before the intervention. Individual interviews with the targeted population revealed that the long-term nature of the exercise rehabilitation process could lead to a decrease in exercise commitment and adherence due to patients’ slackness at a later stage, a phenomenon also reported in the team’s follow-up study. Therefore, timely identification of the emergence of exercise slack needs to be explored in greater depth to develop targeted corrective strategies.

## Appendix

Multimedia Appendix 1 The “SCeiP” model adopted from Dr. Wang, 2021 [24]. SCeiP: Self-Evaluation/Condition of Exercise-Effect Perception-Internal Drive-Persistence Behavior.

Multimedia Appendix 2 CONSORT-EHEALTH (V 1.6.1) checklist. CONSORT: Consolidated Standards of Reporting Trials.

Multimedia Appendix 3 Specific intervention programs for the cognitive phase.

## Abbreviations

CHD: coronary heart disease
CONSORT: Consolidated Standards of Reporting Trials
SCeiP: Self-Evaluation/Condition of Exercise-Effect Perception-Internal Drive-Persistence Behavior

## Data Availability

The research data are uploaded to a publicly accessible personal database [48] with stringent usage restrictions. The study researchers can be contacted for utilization purposes if necessary.

## References

[1] Anderson L J, Taylor R S (2014). Cardiac rehabilitation for people with heart disease: an overview of Cochrane systematic reviews. Int J Cardiol.

[2] Dibben G, Faulkner J, Oldridge N, Rees K, Thompson D, Zwisler A, Taylor Rod S (2023). Exercise-based cardiac rehabilitation for coronary heart disease: a meta-analysis. Eur Heart J.

[3] Bakhshayeh S, Sarbaz M, Kimiafar K, Vakilian F, Eslami S (2021). Barriers to participation in center-based cardiac rehabilitation programs and patients' attitude toward home-based cardiac rehabilitation programs. Physiother Theory Pract.

[4] Thomas RJ, Beatty AL, Beckie TM, Brewer LC, Brown TM, Forman DE, Franklin BA, Keteyian SJ, Kitzman DW, Regensteiner JG, Sanderson BK, Whooley MA (2019). Home-based cardiac rehabilitation: a scientific statement from the American Association of Cardiovascular and Pulmonary Rehabilitation, the American Heart Association, and the American College of Cardiology. Circulation.

[5] Ramachandran H, Jiang Y, Tam W, Yeo T, Wang W (2022). Effectiveness of home-based cardiac telerehabilitation as an alternative to phase 2 cardiac rehabilitation of coronary heart disease: a systematic review and meta-analysis. Eur J Prev Cardiol.

[6] Antoniou V, Kapreli E, Davos CH, Batalik L, Pepera G (2024). Safety and long-term outcomes of remote cardiac rehabilitation in coronary heart disease patients: a systematic review. Digit Health.

[7] Grace SL, Turk-Adawi K, Santiago de Araújo Pio Carolina, Alter DA (2016). Ensuring cardiac rehabilitation access for the majority of those in need: a call to action for Canada. Can J Cardiol.

[8] Abreu A, Mendes M, Dores H, Silveira C, Fontes P, Teixeira M, Santa Clara H, Morais J (2018). Mandatory criteria for cardiac rehabilitation programs: 2018 guidelines from the Portuguese Society of Cardiology. Rev Port Cardiol (Engl Ed).

[9] Ruano-Ravina A, Pena-Gil C, Abu-Assi E, Raposeiras S, van 't Hof A, Meindersma E, Bossano Prescott EI, González-Juanatey Jose Ramón (2016). Participation and adherence to cardiac rehabilitation programs. A systematic review. Int J Cardiol.

[10] Sommer CG, Jørgensen Lars Bo, Blume B, Møller Tom, Skou ST, Harrison A, Tang LH (2022). Dropout during a 12-week transitional exercise-based cardiac rehabilitation programme: a mixed-methods prospective cohort study. Eur J Cardiovasc Nurs.

[11] British Heart Foundation (2018). The National Audit of Cardiac Rehabilitation: Quality and Outcomes Report 2018. British Heart Foundation.

[12] Cabrera-Aguilera I, Ivern C, Badosa N, Marco E, Salas-Medina L, Mojón Diana, Vicente M, Llagostera M, Farré Nuria, Ruiz-Bustillo S (2021). Impact of and reasons for not performing exercise training after an acute coronary syndrome in the setting of an interdisciplinary cardiac rehabilitation program: results from a risk-op-acute coronary syndrome ambispective registry. Front Physiol.

[13] Santiago de Araújo Pio Carolina, Chaves G, Davies P, Taylor R, Grace S (2019). Interventions to promote patient utilisation of cardiac rehabilitation. Cochrane Database Syst Rev.

[14] Terada T, Vidal-Almela S, Tulloch H, Pipe A, Reed J (2021). Cardiac rehabilitation following percutaneous coronary intervention is associated with superior psychological health and quality of life in males but not in females. J Cardiopulm Rehabil Prev.

[15] Taylor JL, Holland DJ, Keating SE, Leveritt MD, Gomersall SR, Rowlands AV, Bailey TG, Coombes JS (2020). Short-term and long-term feasibility, safety, and efficacy of high-intensity interval training in cardiac rehabilitation: the FITR Heart Study randomized clinical trial. JAMA Cardiol.

[16] Sumner J, Grace SL, Doherty P (2016). Predictors of cardiac rehabilitation utilization in England: results from the national audit. J Am Heart Assoc.

[17] Yin Y, He Q, Zhang R, Cheng H, Zhang Y, Zhang J (2022). Predictors of adherence of enhanced external counterpulsation in patients with coronary heart disease after discharge: a mixed-methods study. Front Cardiovasc Med.

[18] Johnson NA, Heller RF (1998). Prediction of patient nonadherence with home-based exercise for cardiac rehabilitation: the role of perceived barriers and perceived benefits. Prev Med.

[19] Ueland Ø, Gunnlaugsdottir H, Holm F, Kalogeras N, Leino O, Luteijn J, Magnússon S H, Odekerken G, Pohjola M, Tijhuis M, Tuomisto J, White B, Verhagen H (2012). State of the art in benefit-risk analysis: consumer perception. Food Chem Toxicol.

[20] Fogg BJ (2009). A behavior model for persuasive design. Proceedings of the 4th International Conference on Persuasive Technology.

[21] Galloway RD (2003). Health promotion: causes, beliefs and measurements. Clin Med Res.

[22] McAlexander JH, Kim SK, Roberts SD (2015). Loyalty: the influences of satisfaction and brand community integration. Journal of Marketing Theory and Practice.

[23] Holdgaard A, Eckhardt-Hansen C, Lassen C, Kjesbu I, Dall C, Michaelsen K, Sibilitz Kirstine Lærum, Prescott Eva, Rasmusen Hanne Kruuse (2023). Cognitive-behavioural therapy reduces psychological distress in younger patients with cardiac disease: a randomized trial. Eur Heart J.

[24] Wang L (2021). Research on the influencing factors and promoting strategies of college students' exercise persistence behavior. Master's dissertation, Shandong University. CNKI.

[25] Schulz KF, Altman DG, Moher D, CONSORT Group (2010). CONSORT 2010 statement: updated guidelines for reporting parallel group randomised trials. BMJ.

[26] Eysenbach Gunther, CONSORT-EHEALTH Group (2011). CONSORT-EHEALTH: improving and standardizing evaluation reports of Web-based and mobile health interventions. J Med Internet Res.

[27] Pan YS, Jin AM, Wang MX (2022). Methods and common pitfalls of sample size estimation in clinical studies. Chinese Journal of Stroke.

[28] Chinese Medical Association, Chinese Medical Journals Publishing House, Chinese Society of General Practice, Prevention Committee of Chinese Society of Cardiology, Cardiac Rehabilitation Committee of Chinese Society of Cardiology, Editorial Board of Chinese Journal of General Practitioners of Chinese Medical Association (2024). Guideline for primary care of cardiac rehabilitation of coronary artery disease (2020). Chinese Journal of General Practitioners.

[29] Micklewright D, Northeast L, Parker P, Jermy M, Hardcastle J, Davison R, Sandercock Gavin, Shearman Jeremy (2016). The Cardiac Rehabilitation Inventory: a new method of tailoring patient support. J Cardiovasc Nurs.

[30] Wang JH, Zhang ZX, Yang QF, Mei YX, Wang P (2019). Translation and reliability and validity of the Chinese version of the Cardiac Rehabilitation Inventory. Chinese Journal of Nursing.

[31] Shen MY (2011). Intervention strategies of Chinese adults’ exercise behavior: the integration of the TPB with the HAPA. Master's dissertation. Beijing Sport University. Dissertation.

[32] Dong BL (2017). Physical exercise involvement of college students in China: measurement, antecedent and aftereffect. Journal of Tianjin University of Sport.

[33] Schmitz B, Wirtz S, Sestayo-Fernández Manuela, Schäfer Hendrik, Douma ER, Alonso Vazquez M, González-Salvado Violeta, Habibovic M, Gatsios D, Kop WJ, Peña-Gil Carlos, Mooren F (2024). Living lab data of patient needs and expectations for eHealth-based cardiac rehabilitation in Germany and Spain from the TIMELY study: cross-sectional analysis. J Med Internet Res.

[34] Su JJ, Yu DS (2021). Effects of a nurse-led eHealth cardiac rehabilitation programme on health outcomes of patients with coronary heart disease: a randomised controlled trial. Int J Nurs Stud.

[35] Akiash N, Mohammadi M, Mombeini H, Nikpajouh A (2021). Myocardial strain analysis as a non-invasive screening test in the diagnosis of stable coronary artery disease. Egypt Heart J.

[36] Chu JK, Zhao Q, Men L, Zhou XR, Li XM, Li QJ, Song N, Liang CY, Yang YN (2023). Application of the new diagnosis and treatment model of internet+medical union based on wearable ECG equipment in cardiovascular disease. Chinese Journal of Cardiovascular Research.

[37] Yu T, Xu H, Sui X, Zhang X, Pang Y, Yu T, Lian X, Zeng T, Wu Y, Leng X, Li F (2023). Effectiveness of eHealth interventions on moderate-to-vigorous intensity physical activity among patients in cardiac rehabilitation: systematic review and meta-analysis. J Med Internet Res.

[38] Guo WT, Liu JP, Zhang XX, Zhang BB, Wu YZ, Wang WJ (2023). Effectiveness and compliance of remote cardiac rehabilitation：an overview of systematic reviews. Chinese Journal of Nursing.

[39] Nabutovsky I, Ashri S, Nachshon A, Tesler R, Shapiro Y, Wright E, Vadasz B, Offer A, Grosman-Rimon L, Klempfner R (2020). Feasibility, safety, and effectiveness of a mobile application in cardiac rehabilitation. Isr Med Assoc J.

[40] Cao Q, Xu L, Wen S, Li F (2021). Investigating the influence of the shared decision-making perception on the patient adherence of the home- and exercise-based cardiac rehabilitation after percutaneous coronary intervention. Patient Prefer Adherence.

[41] Sun L, Wu T, Zhang M, Huang S, Zeng Z, Wu Y (2022). Investigation on family support system and willingness of patients to participate in cardiac rehabilitation after percutaneous coronary intervention. Evid Based Complement Alternat Med.

[42] Antoniou V, Davos CH, Kapreli E, Batalik L, Panagiotakos DB, Pepera G (2022). Effectiveness of home-based cardiac rehabilitation, using wearable sensors, as a multicomponent, cutting-edge intervention: a systematic review and meta-analysis. J Clin Med.

[43] Lahtio H, Heinonen A, Paajanen T, Sjögren Tuulikki (2023). The added value of remote technology in cardiac rehabilitation on physical function, anthropometrics, and quality of life: cluster randomized controlled trial. J Med Internet Res.

[44] Indraratna P, Biswas U, McVeigh J, Mamo A, Magdy J, Vickers D, Watkins E, Ziegl A, Liu H, Cholerton N, Li J, Holgate K, Fildes J, Gallagher R, Ferry C, Jan S, Briggs N, Schreier G, Redmond SJ, Loh E, Yu J, Lovell NH, Ooi S (2022). A smartphone-based model of care to support patients with cardiac disease transitioning from hospital to the community (TeleClinical Care): pilot randomized controlled trial. JMIR Mhealth Uhealth.

[45] Oehler AC, Holmstrand EC, Zhou L, Harzand A, Vathsangam H, Kendall K, Gabriel G, Murali S (2024). Cost analysis of remote cardiac rehabilitation compared with facility-based cardiac rehabilitation for coronary artery disease. Am J Cardiol.

[46] Dwiputra B, Santoso A, Purwowiyoto BS, Radi B, Ambari AM, Desandri DR, Fatrin S, Pandhita BAW (2023). Smartphone-based cardiac rehabilitation program improves functional capacity in coronary heart disease patients: a systematic review and meta-analysis. Glob Heart.

[47] Mitropoulos A, Anifanti M, Koukouvou G, Ntovoli A, Alexandris K, Kouidi E (2024). Exploring the effects of real-time online cardiac telerehabilitation using wearable devices compared to gym-based cardiac exercise in people with a recent myocardial infarction: a randomised controlled trial. Front Cardiovasc Med.

[48] GitHub.

