# Supplementary figures and images for: The Effectiveness of Remote Exercise Rehabilitation Based on the “SCeiP” Model in Homebound Patients With Coronary Heart Disease: Randomized Controlled Trial
Source: J Med Internet Res. 2024 Nov 5;26:e56552. doi: 10.2196/56552 (PMC11576597; doi:10.2196/56552)

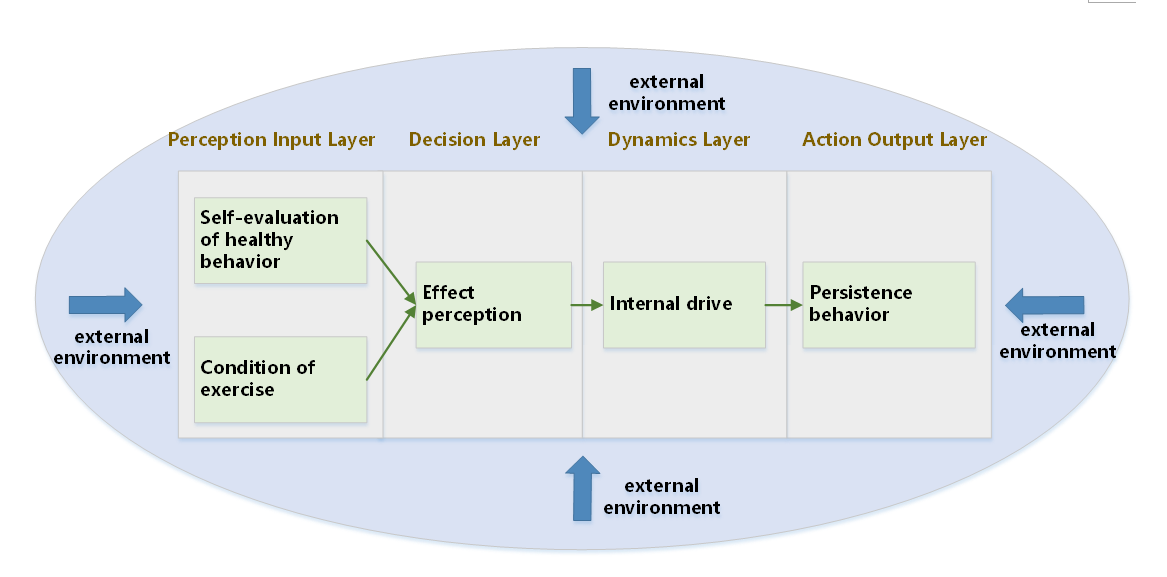

Supplement: Multimedia Appendix 1 [file jmir_v26i1e56552_app1.png]
