# Supplementary material for: The Effectiveness of Remote Exercise Rehabilitation Based on the “SCeiP” Model in Homebound Patients With Coronary Heart Disease: Randomized Controlled Trial
Source: J Med Internet Res. 2024 Nov 5;26:e56552. doi: 10.2196/56552 (PMC11576597; doi:10.2196/56552)
Supplement: Multimedia Appendix 3 [file jmir_v26i1e56552_app3.docx]

Multimedia Appendix 2: Specific intervention programs for the cognitive phase

| Intervention time | Forms of intervention | Details |
| --- | --- | --- |
| First Week | Self-assessment of exercise cognition | (a)Investigate the patients' knowledge of the exercise program and develop appropriate scientific lectures on knowledge related to exercise rehabilitation. |
|  | self- assessment of lifestyle | (a)Collecting patients' lifestyle patterns, with a particular focus on patients' personal exercise habits, customizing health records, and upgrading behavioral records;  (b)Realize the whole environment assessment of exercise rehabilitation through the rational management, analysis and utilization of patients' health information. |
|  | Self-assessment of health status | (a)Record and track patients' biochemical indicators such as cardiac markers, blood glucose levels, blood lipid levels, and other vital signs such as blood pressure and heart rate through rational management, analysis, and utilization of patients' health behavior data in the hospital;  (b) Perform time-series comparisons to form a stable data record of behavioral trends and mark the normal behavioral data change interval. |
|  | Self-assessment  of individual inputs | (a) Assessing the benefits of exercise rehabilitation, the personal inputs and actual benefits of individuals' participation in exercise behaviors, and whether they meet expected expectations |
| Second week | Feedback on self- assessment | (a)Assess the barriers and facilitators to the implementation of exercise-promoting behaviors based on the patient's self- assessment. |
|  | Group health education | (a) To hand over the knowledge of coronary heart disease related diseases and exercise rehabilitation related contents to the patients, so that the patients can realize the benefits of exercise rehabilitation and the risks of not exercising;  (b)Explain disease risk factors to patients, remind patients to avoid risk factors in daily life, improve patients' negative cognition and emotions, and realize the benefits of exercise and exercise safety;  (c)Help patients to understand the knowledge of diseases related to coronary heart disease and the content related to exercise rehabilitation, so that they can recognize the benefits of exercise rehabilitation and the risks of not exercising;  (d)Help patients establish good living habits and encourage them to make appropriate modifications to their exercise living environment. |
|  | Patient Networking Meeting | (a) Guide patients to exchange their experiences and gains in disease treatment and exercise promotion, jointly solve the confusing problems in the upcoming exercise program, and encourage patients to establish regular exercise habits as soon as possible. |
|  | Face-to-face contact | (a)Organize one-on-one meetings between cardiologists or rehabilitation physicians and patients to answer questions and assess exercise tendencies |
